# Conjunctival Scarring, Corneal Pannus, and Herbert’s Pits in Adolescent Children in Trachoma-endemic Populations of the Solomon Islands and Vanuatu

**DOI:** 10.1093/cid/ciaa1151

**Published:** 2020-08-10

**Authors:** Robert Butcher, Junely Tagabasoe, Joseph Manemaka, Annie Bong, Mackline Garae, Lui Daniel, Chrissy Roberts, Becca L Handley, Victor H Hu, Emma M Harding-Esch, Ana Bakhtiari, Rebecca Willis, Andreas Müller, John Kaldor, Richard Le Mesurier, David Mabey, Anasaini Cama, Oliver Sokana, Fasihah Taleo, Hugh R Taylor, Anthony W Solomon

**Affiliations:** 1 Clinical Research Department, London School of Hygiene & Tropical Medicine, London, United Kingdom; 2 Eye Department, Solomon Islands Ministry of Health and Medical Services, Honiara, Solomon Islands; 3 Health Promotion Department, Solomon Islands Ministry of Health and Medical Services, Honiara, Solomon Islands; 4 Eye Department, Vanuatu Ministry of Health, Port Vila, Vanuatu; 5 Department of Neglected Tropical Diseases, Vanuatu Ministry of Health, Port Vila, Vanuatu; 6 Task Force for Global Health, Decatur, Georgia, USA; 7 Centre for Eye Research Australia, University of Melbourne, Melbourne, Australia; 8 Programme for Blindness and Visual Impairment, World Health Organization, Geneva, Switzerland; 9 Kirby Institute, University of New South Wales, Sydney, Australia; 10 The Fred Hollows Foundation, Carlton, Victoria, Australia; 11 Country Office, World Health Organization, Port Vila, Vanuatu Country Office, Vanuatu; 12 Indigenous Eye Health, Melbourne School of Population and Global Health, University of Melbourne, Melbourne, Australia; 13 Department of Control of Neglected Tropical Diseases, World Health Organization, Geneva, Switzerland

**Keywords:** trachoma, conjunctival scarring, corneal pannus, Herbert’s pits

## Abstract

**Background:**

In the Solomon Islands and Vanuatu, the sign trachomatous inflammation—follicular (TF) is common, but ocular infection with *Chlamydia trachomatis* is not. It is therefore debatable whether azithromycin mass drug administration (MDA), the recommended antibiotic treatment strategy for trachoma’s elimination as a public health problem, is necessary in this setting. We set out to estimate what proportion of adolescents were at risk of progression of trachomatous scarring.

**Methods:**

A cross-sectional survey was undertaken of all children aged 10–14 years resident in communities identified as high-TF clusters during previous population-based mapping. Graders examined children for clinical evidence of trachomatous scarring, pannus, and Herbert’s pits (HPs) or limbal follicles in both eyes. A dried blood spot was collected from each child and tested for antibodies to *C. trachomatis*.

**Results:**

A total of 492 children in 24 villages of the Solomon Islands and Vanuatu were examined. In total, 35/492 (7%) of children had limbal signs (pannus and/or HPs) plus any conjunctival scarring. And 9/492 (2%) had limbal signs and moderate or severe conjunctival scarring; 22% of children were anti-Pgp3 seropositive.

**Conclusions:**

Few adolescents here are at risk of future complications from trachoma, supporting the conclusion that further antibiotic MDA is not currently required for trachoma elimination purposes in these settings.

## Teaser

In Solomon Islands and Vanuatu communities in which active trachoma prevalence in children was previously high, prevalence of Herbert’s pits or pannus plus conjunctival scarring in 10–14-year-olds is low, suggesting risk of future trachomatous blindness here is also low.

Trachoma is the most common infectious cause of blindness [1] and a neglected tropical disease targeted for global elimination as a public health problem. Trachoma is considered eliminated as a public health problem when 3 criteria are satisfied in each formerly endemic district: (a) prevalence of trachomatous inflammation—follicular (TF) in 1–9-year-olds <5%; (b) prevalence of trachomatous trichiasis (TT) unknown to the health system in ≥15-year-olds <0.2%; and (c) presence of a system to identify and manage incident cases of TT [2].

TT is caused by conjunctival scarring distorting the eyelid, which is a result of repeated ocular infection with *Chlamydia trachomatis* (*Ct*) and associated inflammation. In trachoma-endemic countries, most adults have some scarring, and ~1% of scarred individuals develop TT each year [3]. Alongside promoting facial cleanliness and improving access to water and sanitation, World Health Organization (WHO) guidelines recommend delivery of multiple annual rounds of azithromycin MDA where the TF prevalence in 1–9-year-olds ≥10% [4]. These interventions are undertaken to reduce *Ct* transmission intensity, to reduce the number of lifetime episodes of infection and disease [5], and hence reduce the risk of today’s children developing conjunctival scarring in later life [6, 7]. In most mapped areas of Melanesia, the TF prevalence in 1–9-year-olds exceeds 10%, but the prevalence of TT is <0.2% [8–11]. Prevalence and transmission of ocular *Ct* are also very low [12, 13]. As a consequence, the question of whether azithromycin MDA for trachoma control is needed in Melanesia has been discussed at length at regional and global levels [14, 15].

One hypothesis for the discrepancy between TF and TT prevalence in Melanesia is that recent increases in population density [16] have driven an increase in TF prevalence. According to this hypothesis, the low TT prevalence indicates that recrudescence is so recent that those exposed to active (inflammatory) trachoma in childhood have not yet lived long enough to develop blinding sequelae. Although empirical data to support this hypothesis are lacking [17], it is important to understand whether the current TF prevalence in 1–9-year-olds is driving conjunctival scarring that might lead to future blindness. If it is, interventions against active trachoma are likely needed; if not, interventions are probably not indicated.

Upper pole corneal pannus and Herbert’s Pits (HPs) (referred to as “limbal signs” in this paper, because both are found at or adjacent to the sclerocorneal junction) are held to be specific, long-lived cicatricial markers of current or previous active trachoma [18–21]; importantly, in one cohort study [22], the presence of ≥2 mm of pannus conferred strong risk of development of severe conjunctival scarring over the course of >15 years follow-up. In another study, presence and severity of pannus was associated with visual impairment [23]. In light of evidence that most observed local TF was unassociated with current or previous ocular *Ct* infection, the prevalence of limbal signs in Melanesia was predicted to be illuminating.

In designing the study, there were a number of additional considerations. First, despite existing data [12, 13] on low prevalence ocular *Ct* infection and low *Ct* transmission intensity in children, some stakeholders remained of the view that MDA was required. Second, there was no established cohort study in Melanesia from which scarring incidence could be estimated. Third, given the multiple competing priorities of public health systems and the general success of the trachoma elimination program elsewhere [24], there was understandable enthusiasm to address the question quickly, enabling the respective programs to move forward. Other potentially useful ways to estimate incidence of scarring or TT, such as a new cohort study, would take years to decades to generate useful data.

Prompted by the recommendations of a WHO expert consultation [14], we set out to investigate whether 10–14-year-olds already had conjunctival scarring and other, more specific limbal signs of previous active trachoma. A moderate-to-high prevalence of the combination of conjunctival scarring plus either of the limbal signs was felt at the WHO expert consultation to signify some risk of future trichiasis that would be unequivocally trachoma-related.

Our aim therefore was to determine the prevalence of pannus, HPs, and conjunctival scarring in children living in villages in which a high proportion of 1–9-year-olds previously had TF. Few contemporary estimates of population prevalence of limbal disease in trachoma-endemic settings have been made [22, 25, 26]; therefore, for the purposes of this study, data from Australia [23] were used to set an a priori threshold for abnormal prevalence and hence continuation/cessation of MDA. As ocular *Ct* infection is a key risk factor for incidence and progression of conjunctival scarring, the study also collected capillary blood specimens for assessment of anti-*Ct* antibodies to determine what proportion of the adolescent children had previously been exposed to infection.

## METHODS

### Study Ethics, Consent, and Data Management

Protocols were approved by the London School of Hygiene & Tropical Medicine (LSHTM) Ethics Committee (15262), the Solomon Islands National Health Research Ethics Committee (12/06/18), the Vanuatu Ministry of Health Ethics Board (DPH 06/17/2-LT/mg) and the Ethics Review Committee of the WHO Regional Office for the Western Pacific (2018.4.VAN.1.CSU).

Village heads provided verbal consent to enroll villages, and household heads provided verbal consent to enroll households. Each participant’s parent or guardian provided written consent, and participants themselves provided verbal assent. Participants with trachoma were offered treatment in accordance with national guidelines.

Data were collected using smartphone-based electronic data collection forms, encrypted and uploaded to secure cloud storage, hosted by Tropical Data (www.tropicaldata.org).

### Study Population

**Figure 1.**
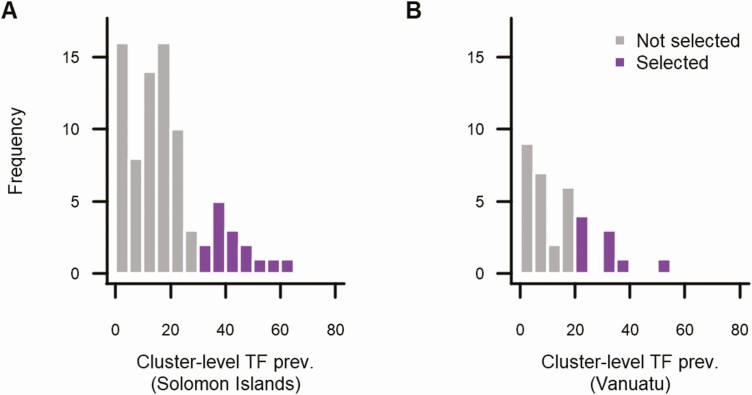
Age- and sex-adjusted cluster-level TF prevalence in 1–9-year-olds from pre-MDA programmatic mapping data in the (*A*) Solomon Islands [9] and (*B*) Vanuatu [27]. Abbreviation: TF, trachomatous inflammation—follicular.

We sought villages where high proportions of 1–9-year-olds had previously had TF [9, 27]. Thresholds for inclusion were set arbitrarily at 20% and 30% TF in Vanuatu and Solomon Islands, respectively [14]. In pre-MDA trachoma surveys in Melanesia, each cluster was an individual village [9, 27]. Villages for inclusion here were therefore identified by extracting age- and sex-adjusted cluster-level TF prevalences from the pre-MDA programmatic survey data [9, 27]. Figure 1 shows histograms of cluster-level pre-MDA TF prevalence in Vanuatu and Solomon Islands and indicates the fractions selected for inclusion.

In Vanuatu, the pre-MDA survey cluster-level TF prevalence in 1–9-year-olds was ≥20% in 11 villages in June–July 2016 [27]. Of these villages, 2 were excluded due to activity of the Manaro Voui volcano on nearby Ambae and resultant evacuation [28]. The mean cluster-level TF prevalence in 1–9-year-olds during pre-MDA mapping in the remaining nine selected villages was 29% (range: 20–51%).

In Temotu and Western provinces, Solomon Islands, the pre-MDA survey cluster-level TF prevalence in 1–9-year-olds was ≥30% in 16 villages in September–November 2013 [9]. One of these villages was excluded because it would have taken a disproportionately long time to revisit. The mean cluster-level TF prevalence in 1–9-year-olds during pre-MDA mapping in the remaining 15 selected Solomon Islands villages was 46% (range: 30–64%).

Children aged 10–14 years living in each selected village were recruited because, in the hypothesized scenario of re-emergence, these individuals would have had time to develop scarring since the pre-MDA surveys of 1–9-year-olds. Teams went house-to-house to register all 10–14-year-olds (both present and absent). They also recorded total numbers of household residents. Where a child was temporarily absent, teams made a return visit to the household or, where possible, visited the child’s school.

### Examination

Elements from 3 established trachoma grading systems were employed (Table 1). Presence and severity of tarsal conjunctival scarring were assessed using the 1981 modified WHO grading scheme [29]. For assessment of pannus and HPs, a modified subset of the “minimal examination” criteria from the 1966 WHO trachoma grading scheme [30] was used. For simplicity, active and inactive pannus were not differentiated, inflammatory corneal infiltration was not graded, and current limbal follicles were graded as HPs.

**Table 1.** Grading Schemes Used in Field and Photographic Grading in This Study

Feature	Degree of Involvement	Grade
Pannus (measured vertically from the upper limbus)	<2.0 mm extension	0
	2.0 to <4.0 mm extension	1
	4.0 to <6.0 mm extension	2
	≥6.0 mm extension	3
Herbert’s pits	None	0
	1–3	1
	>3 but not involving the entire upper lunular	2
	Entire upper lunular involved	3
	Cornea encircled or 2 rows of pits above	4
Cicatricae	None	0
	Fine scattered scars on the upper tarsal conjunctiva, or scars on other parts of the conjunctiva (mild)	1
	More severe scarring but without shortening or distortion of the upper tarsus (moderate)	2
	Scarring with distortion of the upper tarsus (severe)	3
Conjunctival inflammation	≥5 follicles each ≥ 0.5 mm diameter in the central part of the upper tarsal conjunctiva	TF
	Pronounced inflammatory thickening of the upper tarsal conjunctiva that obscures more than half of the normal deep tarsal vessels	TI

Grading systems taken directly from [29, 31] and modified from [30].

Abbreviations: TF, trachomatous inflammation—follicular; TI, trachomatous inflammation—intense.

Field examinations were carried out by ophthalmic nurses (field graders). Two months before the survey, the field graders undertook a 2-day training program on the grading of conjunctival scars, pannus, and HPs; they then participated in a 1-day refresher course immediately before the survey started. These training sessions involved presentation of photographs of typical cases, discussion of key features and an assessment of photographs. For the first week of fieldwork, all field graders were supervised in the field by experienced trachoma graders.

### Photography

The objective for collecting photographs was to determine their utility for future studies by comparing agreement with field grades. High-resolution digital photographs were taken of the conjunctiva and upper pole of the limbus of each eye. Low quality and obscured photographs were discarded prior to random selection for grading. A random sample of 60 conjunctival and 60 limbus photographs was graded by 2 independent ophthalmologists with substantial experience of trachoma grading. Two analyses were conducted. First, field grades on scarring, pannus and HPs were compared to photograph grades independently assigned by photograder (PG) 1 and PG2 using weighted kappa scores. Second, the two PGs met to discuss photographs for which a photograph grade discrepancy had occurred. For this discussion, PGs were provided with their own independent grades and the field grades and asked to come to a consensus as to whether there was any scarring (C ≥1) in a conjunctival photograph or any limbal disease (pannus ≥2 mm or ≥1 HP) in a limbal photograph. This consensus grade was then compared to the field grade. Where either PG felt a grade could not be assigned due to lack of image clarity, they were omitted from the comparison.

### Serological Testing

Blood was collected, dried, and stored as described previously [31]. Dried blood spots (DBS) were shipped to LSHTM and tested for anti-Pgp3 antibodies using the Luminex system. The assay used was modified from Goodhew et al. [32] by utilizing a secondary antibody with prebound streptavidin rather than adding secondary antibody and streptavidin in separate steps. A serial dilution of high-titer serum, low-titer serum, and buffer without serum were run in triplicate as controls on each plate. The threshold net median fluorescence intensity (MFI) for seropositivity was determined as 815 by receiver–operator curve analysis against a panel of previously characterized positive (n = 34) and negative (n = 35) samples [32].

### Data Analyses

Primary outcome measures were the prevalence of defined combinations of signs in 10–14-year-olds, as prespecified by the WHO expert consultation: (i) limbal signs (pannus, HPs, or both) in at least 1 eye plus conjunctival scarring (C1, C2, or C3) in at least 1 eye; and (ii) limbal signs in at least 1 eye and moderate-to-severe conjunctival scarring (C2 or C3) in at least 1 eye. Because a grader in the field can examine the eye in 3 dimensions for as long as needed, participant-willing, these outcome measures were based on the field grade.

The relationship between any limbal sign with age, sex, TF, country of residence, anti-Pgp3 serostatus, and presence of scarring was tested using mixed-effects logistic regression (lme4::glmer function in R). Village of residence was included as a random effect in all models to account for clustering of trachoma at village level. For each independent fixed-effect variable to be included in the multivariable mixed-effects model, it had to be (a) associated with limbal signs in univariate analysis (*P* < .1) and (b) not strongly correlated with another independent variable (polycor::hetcor function in R). Significance of association was tested with likelihood ratios.

## RESULTS

### Study Population

We visited 24 villages. We recruited and examined 492 residents aged 10–14 years, constituting 93% of individuals in that age group living in selected villages during the study (Table 2). Of 41 nonresponders, 34 (83%) were absent on the day of examination, 2 (5%) refused, and specific reasons for nonparticipation were not recorded for 5.

**Table 2.** Study Population Demographics During Survey of Upper Pole Corneal Pannus, Herbert’s Pits, and Conjunctival Scarring in Selected Villages of the Solomon Islands and Vanuatu, 2018

	Solomon Islands	Vanuatu	Total
Survey month	June–July 2018	August 2018	June–August 2018
Villages visited/villages targeted	15/16	9/11	24/27
Households enrolled	217	133	350
Household residents aged ≥1 year	1128	835	1963
Household residents aged 10–14 years	332	197	529
Residents surveyed aged 10–14 years	323 (97%)	169 (86%)	492 (93%)
Male 10–14-year-olds surveyed (%)	51	53	52
Median (range) 10–14-year-olds per village	21 (8–37)	15 (7–42)	18 (7–42)
Median (range) individuals per 1-year age band	66 (52–73)	33 (25–48)	99 (80–121)

### Clinical Examination

Examination results are in Table 3. A larger proportion of 10–14-year-olds in the Solomon Islands had TF than in Vanuatu (83/323 [26%] vs 8/169 [5%]; Welch *t*-test *P* < .001). The proportion of participants with a scarring grade of C ≥ 1 in at least one eye was higher in the Solomon Islands than in Vanuatu (112/323 [35%] vs 18/169 [11%]; Welch *t*-test *P* < .001). Of those with scarring in at least 1 eye, 54/130 (42%) had scarring in 1 eye but not the other. A further 12/130 (9%) individuals with scarring had 2 scarred eyelids, which differed in scarring severity. Scarring was not associated with sex or age but was less common in participants with TF (ordered logistic regression *P* = .02).

**Table 3.** Upper Pole Corneal Pannus and Herbert’s Pits in Children aged 10–14 Years in Selected Villages of the Solomon Islands (n = 323) and Vanuatu (n = 169), 2018

		Solomon Islands (%)	Vanuatu (%)	Total (%)
**Pannus**	**0**	283 (88)	148 (88)	431 (88)
	**1**	40 (12)	21 (12)	61 (12)
	**2**	0 (0)	0 (0)	0 (0)
	**3**	0 (0)	0 (0)	0 (0)
**Herbert’s pits**	**0**	281 (87)	160 (95)	441 (90)
	**1**	36 (11)	9 (5)	4 (9)
	**2**	5 (2)	0 (0)	5 (1)
	**3**	1 (0.3)	0 (0)	1 (0.2)
	**4**	0 (0)	0 (0)	0 (0)
**Scarring**	**C0**	211 (65)	151 (89)	362 (74)
	**C1**	86 (27)	16 (9)	102 (21)
	**C2/C3**	26 (8)	2 (1)	28 (6)
**TF**		83 (26)	8 (5)	91 (18)
**TI**		4 (1)	0 (0)	4 (1)

Abbreviations: TF, trachomatous inflammation—follicular; TI, trachomatous inflammation—intense.

In total, 87/492 (18%) children had limbal signs in at least 1 eye (Table 3). In total, 47/61 (77%) cases of pannus, and 24/51 (47%) cases of HPs were bilateral. Limbal signs were more common in people with TF (adjusted odds ratio [aOR]: 4.30, 95% confidence interval [CI]: 2.25–8.22, *P* < .001; Supplementary Table 1) and also more common in those with conjunctival scarring (aOR: 2.30, 95% CI: 1.28–4.14, *P* = .006; Supplementary Table 1). Limbal signs were less common in females (aOR: 0.48, 95% CI: .28–.82, *P* = .007; Supplementary Table 1). Neither serostatus nor age were independently associated with limbal signs (Supplementary Table 1).

### Scars and Limbal Signs

**Figure 2.**
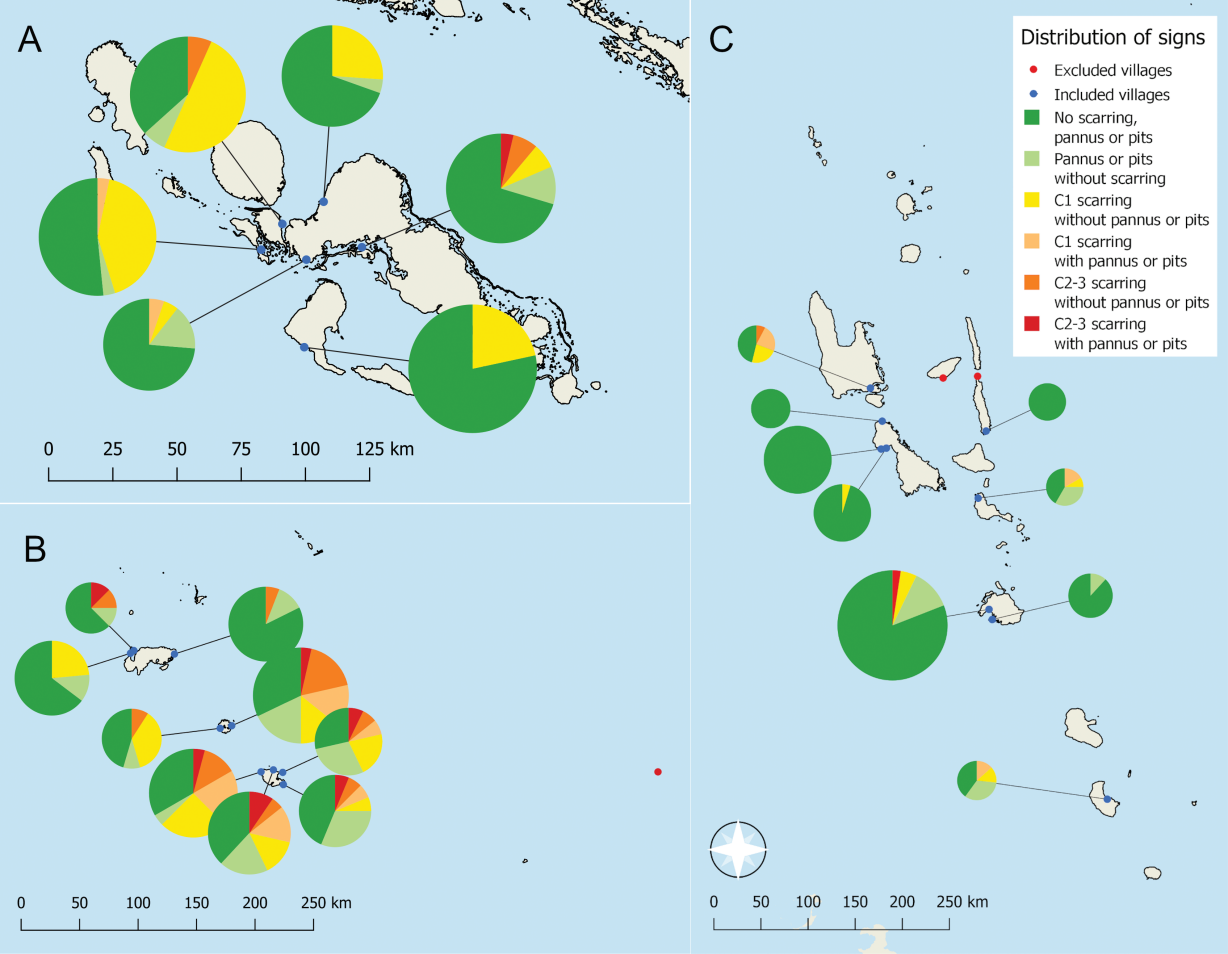
Geographical distribution of upper pole corneal pannus, Herbert’s pits and conjunctival scarring by village in (*A*) Western province, Solomon Islands, (*B*) Temotu province, Solomon Islands, and (*C*) Vanuatu. Surveys conducted in 2018. The area of each pie chart is proportional to the number of children examined in that village. The legend category is determined at the individual level.

In total, 35/492 (7%) children had both a limbal sign and any conjunctival scarring (C ≥ 1). And 9/492 (2%) had both a limbal sign and moderate-to-severe conjunctival scarring (C ≥ 2) (Table 4). Cases of moderate-to-severe scars and limbal signs were not evenly distributed within either country. The proportions of children affected in each visited village in the Solomon Islands and Vanautu are shown in Figure 2.

**Table 4.** Concurrence of Upper Pole Corneal Pannus, Herbert’s Pits, and Conjunctival Scars at the Individual Level in Children Aged 10–14 Years, in Selected Villages of the Solomon Islands (n = 323) and Vanuatu (n = 169), 2018.

		Herbert’s pits (HPs) and/or Pannus (Most Severely Affected Eye)			
Conjunctival Scar Grade (Most Severely Affected Eye)		No (%)	Yes (%)	Primary Outcome (%)^a^	
Solomon Islands	C0	175 (54)	36 (11)		
	C1	68 (21)	18 (6)	**26 (8.0)**	
	C2	15 (5)	5 (2)		**8 (2.5)**
	C3	3 (1)	3 (1)		
Vanuatu	C0	135 (80)	16 (9)		
	C1	8 (5)	8 (5)	**9 (5.3)**	
	C2	0 (0)	0 (0)		**1 (0.6)**
	C3	1 (1)	1 (1)		

Study Outcomes are Shown in Bold Font.

^a^The primary outcomes of the study were the prevalence of defined combinations of signs in 10–14-year-olds, specifically (i) limbal signs (pannus, HPs, or both) in at least one eye plus conjunctival scarring (C ≥ 1) in at least 1 eye; and (ii) limbal signs (pannus, HPs, or both) in at least 1 eye and moderate-to-severe conjunctival scarring (C ≥ 2) in at least 1 eye.

### Photograph Grading

The intergrader agreement between PG1, PG2, PG concensus, and field grade is shown in Table 5. In general, PGs called more cases of limbal features and fewer cases of conjunctival scarring than field graders.

**Table 5.** Agreement Between Field and Photograph Grading

Grade	Comparison	Field − Photo −	Field − Photo +	Field + Photo −	Field + Photo +	Percent Agreement	Kappa Score
Any scar (C ≥ 1)	Field vs PG1 (n = 60)	32	14	6	8	67	0.22
	Field vs PG2 (n = 58)	43	1	9	5	83	0.42
	Field vs PG consensus (n = 53)	39	2	8	4	81	0.69
Any limbal feature (pannus ≥2 mm and/or Herbert’s pits)	Field vs PG1 (n = 59)	42	8	4	5	80	0.34
	Field vs PG2 (n = 60)	34	17	6	3	62	0.00
	Field vs PG consensus (n = 57)	44	5	1	7	90	0.35

Abbreviation: PG, photograder.

### Anti-Pgp3 Serology

**Figure 3.**
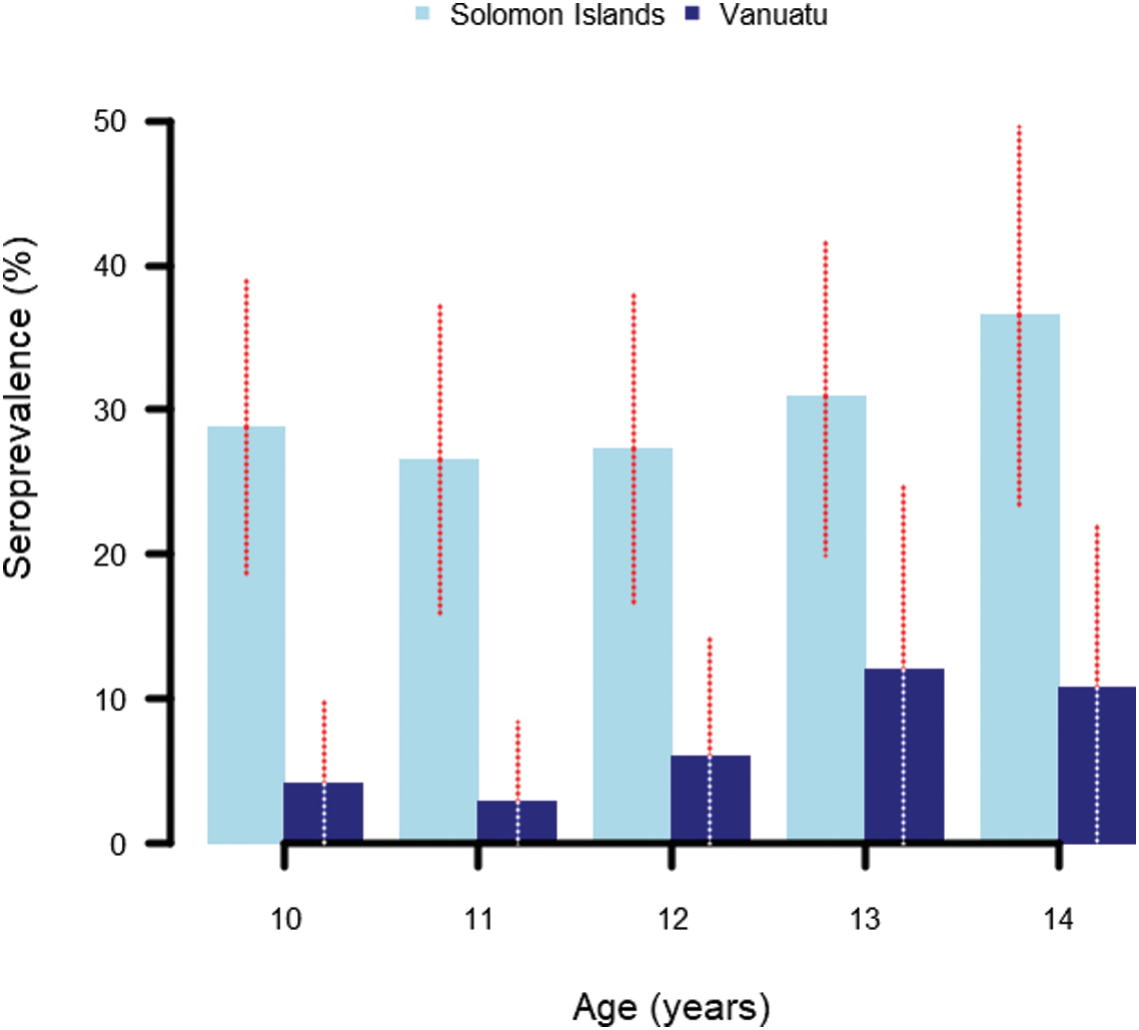
Age-specific anti-Pgp3 seroprevalence in children aged 10–14 years in selected villages of (*A*) Solomon Islands (n = 323) and (*B*) Vanuatu (n = 169), 2018. Whiskers represent Wald’s interval around the seroporevalence estimate at the 95% confidence level.

The age-specific anti-Pgp3 seroprevalence is shown in Figure 3. In the Solomon Islands, 30% (96/323) of participants had antibody levels in excess of the seropositivity threshold. In Vanuatu, 7% (11/169) had antibody levels in excess of the threshold. There was no strong evidence for an increase in seropositivity with age in either country (logistic regression: *P* = .117 and *P* = .053).

## DISCUSSION

Trachoma makes only a very small contribution to the overall burden of blindness and visual impairment in the Western Pacific Region [33], and it is therefore apposite to consider whether WHO-recommended interventions for eliminating trachoma as a public health problem are locally relevant. We found the prevalence of pannus and/or HPs plus conjunctival scarring in 10–14-year-olds in Vanuatu and the Solomon Islands to be <20%, below a predefined threshold for continuation of antibiotic MDA for trachoma [14].

Based on data on limbal signs in trachoma-endemic communities in Guinea Bissau and Taiwan, if TF in the Solomon Islands and Vanuatu was genuinely trachomatous, we could expect 60–80% of those with TF and 20–40% of those without active trachoma to have pannus and/or HPs [21, 25]; the prevalence in these villages is demonstrably lower than that. In one previous study [22], ~90% of individuals with pannus had some degree of conjunctival scarring; in our study, 27% of those with limbal signs had concurrent scarring, suggesting that what we are observing in the Solomon Islands and Vanuatu is unlikely to be blinding trachoma. Previous data from Melanesia have shown a low prevalence of ocular *Ct* infection, a low intensity of *Ct* transmission among children, a relatively low prevalence of scars, and rare-to-absent TT in adults [9, 11, 12, 27]. Together with the current data on limbal disease, it could be concluded that the priority for addressing trachoma in these islands would be to focus on attempting to maintain low *Ct* transmission through facial cleanliness and improving hygiene facilities rather than reducing *Ct* prevalence through antibiotic distribution.

This survey was intended to generate data to address a specific question to guide policy making in the Pacific [14]. The study design had several limitations. First, the identification of villages for inclusion was opportunistic and based on available data from cluster-sampled surveys rather than a complete list of villages in each evaluation unit. Other hot-spots of disease could exist outside the villages included here. Second, it is clear from the comparison of field to photograph grades that grading of scarring, pannus, and HPs can be inconsistent; similar questions have previously been identified in the use of photographs for diagnosing signs of active trachoma [34, 35]. Third, we did not directly test for ocular *Ct* infection in this study. This was because extensive infection testing had already been done in Solomon Islands and Vanuatu, and the prevalence of infection was known to be low in young children (~1% of 1–9 year olds in the most recent surveys in Temotu and Vanuatu [12, 27]), who are at highest risk of infection [36]. In addition, as trachomatous scarring often develops and worsens in older people in the absence of ongoing *Ct* infection [37, 38], presence or absence of infection in our study subjects was not felt to be predictive of future cicatricial disease. Fourth, antibodies to Pgp3 cannot distinguish between exposure to ocular or urogenital strains; the possibility that some of our seropositive children had seroconverted due to exposure to nonocular strains cannot be ruled out. In fact, due to exposure to *Ct* in mothers’ urogenital tracts at parturition [39, 40–42], ~10% of 1-year-olds in the Solomon Islands [13] and ~5% of 1-year-olds in Vanuatu [27] are seropositive. Our observed seroprevalence is therefore unlikely to be exclusively attributable to ocular exposure. This supports the hypothesis that transmission of ocular *Ct* is low in these communities. Finally, there are limited comparator data on pannus and HPs and their distribution in trachoma-endemic settings. Although these 2 signs are said to be pathognomonic (or at least highly specific) for trachoma [22], empirical data to demonstrate their specificity are lacking.

In light of these methodological limitations and the specialized nature of the expertise required for its application, we do not recommend surveys of scarring and limbal signs for routine application in assessing need for interventions against active trachoma. Further research should be conducted on pannus and HPs to better characterize the significance of these signs. In the meantime, however, these data suggest that antibiotic MDA is not needed for trachoma elimination purposes in the Solomon Islands or Vanuatu.

## Supplementary Material

ciaa1151_suppl_Supplementary_Table_1

## Notes

***Author contributions.*** Study conception and design: R. B., A. M., J. K., R. L. M., D. M., A. C., H. R. T., A. W. S. Study planning and implementation: R. B., C. R., B. L. H., E. M. H-E., A. Ba., R. W., A. M., R. L. M., A. C., O. S., F. T., A. W. S. Training: R. B., J. T., J. M., B. L. H., R. L. M., A. C., H. R. T., A. W. S. Data collection: R. B., J. T., J. M., A. Bo., M. G., L. D., B. L. H., V. H. H., A. C., O. S., F. T Data curation and analysis: R. B., E. M. H-E., R. W., A. Ba. Report preparation: R. B. Report revision and approval: R. B., J. T., J. M., A. Bo., M. G., L. D., C. R., B. L. H., V. H. H., E. M. H-E., A. Ba., R. W., A. M., J. K., R. L. M., D. M., A. C., O. S., F. T., H. R. T., A. W. S.

***Acknowledgements*.** The authors thank the residents of the Solomon Islands and Vanuatu who took part in the study; the field teams in Vanuatu (Bruce Obed, Kalo Berry, Prudence Rymil) and the Solomon Islands (Chillion Evans, Eric Suri, Annie Rofeta, and Nigel Telikimaoli) who helped collect the data; and Diana Martin and Sarah Gwyn (US Centers for Disease Control and Prevention) who provided reagents for the anti-Pgp3 Luminex assay.

***Financial support*.** Fieldwork was funded by The Fred Hollows Foundation. Laboratory work and the salaries of R. B. and B. L. H. were funded by the Fred Hollows Foundation, the Queen Elizabeth Diamond Jubilee Trust and the International Trachoma Initiative (ITI) through a collaborative award managed by The Fred Hollows Foundation [1954–0]. E. M. H-E. and A. Ba. are funded by the International Trachoma Initiative. ITI is a program of The Task Force for Global Health and receives funding from Pfizer Inc. Neither ITI nor Pfizer Inc. had any role in the study’s design; in the collection, analysis, and interpretation of data; in the writing of the publication; or in the decision to submit for publication.

***Potential conflicts of interest.*** The authors: No reported conflicts of interest. All authors have submitted the ICMJE Form for Disclosure of Potential Conflicts of Interest.
