# Supplementary material for: Conjunctival Scarring, Corneal Pannus, and Herbert’s Pits in Adolescent Children in Trachoma-endemic Populations of the Solomon Islands and Vanuatu
Source: Clin Infect Dis. 2020 Aug 10;73(9):e2773–80. doi: 10.1093/cid/ciaa1151 (PMC8563182; doi:10.1093/cid/ciaa1151)
Supplement: ciaa1151_suppl_Supplementary_Table_1 [file ciaa1151_suppl_supplementary_table_1.docx]

**Supplementary Table 1.** Univariable and multivariable mixed-effects logistic regression of the relationship between having upper pole corneal pannus and/or Herbert’s pits in either eye (i.e., any limbal sign) and age, gender, anti-Pgp3 serostatus, presence of TF and presence of conjunctival scars in children aged 10–14 years (n=492), in selected villages of the Solomon Islands and Vanuatu, 2018. Confidence intervals around odds ratios are calculated using Wald’s test. *P*-values suggesting strong evidence (*p*<0.05), calculated by likelihood ratio testing of models with and without each independent variable, are indicated in **bold**.

| **Variable** | | **Univariable analysis** | | **Multivariable analysis** | |
| --- | --- | --- | --- | --- | --- |
|  |  | **OR**  **(95% CI)** | **p-value** | **aOR**  **(95% CI)** | **p-value** |
| Age (years) | 10–12 | - | - | Not tested | |
|  | 13–14 | 0.86  (0.51–1.47) | 0.593 |  |  |
| Gender | Male | - | - | - | **-** |
|  | Female | 0.50  (0.30–0.83) | 0.008 | **0.48**  **(0.28–0.82)** | **0.007** |
| TF in either eye | No | - | - | - | **-** |
|  | Yes | 3.42  (1.86–6.31) | <0.001 | **4.30**  **(2.25–8.22)** | **<0.001** |
| Any scar (C≥1 in either eye) | No | - | - | - | - |
|  | Yes | 1.85  (1.06–3.23) | 0.034 | **2.30**  **(1.28–4.14)** | **0.006** |
| Country | Solomon Islands | - | - | Not tested | |
|  | Vanuatu | 0.62  (0.20–1.89) | 0.394 |  |  |
| Anti-Pgp3 seropositivity | No | - | - | Not tested | |
|  | Yes | 1.06  (0.54–2.10) | 0.858 |  |  |
| aOR: adjusted odds ratio; C: conjunctival scar graded according to [30]; CI: confidence interval; OR: odds ratio; TF: trachomatous inflammation—follicular. | | | | | |
